# New Described Dermatological Disorders

**DOI:** 10.1155/2014/616973

**Published:** 2014-08-28

**Authors:** Müzeyyen Gönül, Bengu Cevirgen Cemil, Havva Ozge Keseroglu, Havva Kaya Akis

**Affiliations:** Dermatology Clinic, Ankara Dışkapı Yıldırım Beyazıt Education and Research Hospital, Dışkapı, 06110 Ankara, Turkey

## Abstract

Many advances in dermatology have been made in recent years. In the present review article, newly described disorders from the last six years are presented in detail. We divided these reports into different sections, including syndromes, autoinflammatory diseases, tumors, and unclassified disease. Syndromes included are “circumferential skin creases Kunze type” and “unusual type of pachyonychia congenita or a new syndrome”; autoinflammatory diseases include “chronic atypical neutrophilic dermatosis with lipodystrophy and elevated temperature (CANDLE) syndrome,” “pyoderma gangrenosum, acne, and hidradenitis suppurativa (PASH) syndrome,” and “pyogenic arthritis, pyoderma gangrenosum, acne, and hidradenitis suppurativa (PAPASH) syndrome”; tumors include “acquired reactive digital fibroma,” “onychocytic matricoma and onychocytic carcinoma,” “infundibulocystic nail bed squamous cell carcinoma,” and “acral histiocytic nodules”; unclassified disorders include “saurian papulosis,” “symmetrical acrokeratoderma,” “confetti-like macular atrophy,” and “skin spicules,” “erythema papulosa semicircularis recidivans.”

## 1. Introduction

Novel cutaneous entities have been continually reported in the dermatology literature. This paper reviews newly described dermatological disorders. MEDLINE (2008–2014) and Google searches were conducted, using the terms (new described skin disease OR new described cutaneous disorder) AND (new entity in dermatology) AND (novel described skin disorder). Fifteen new entities are included in this paper. Dermatological disorders are classified in one of the following categories: syndromes, tumors, keratinization disorders, and unclassified disease, respectively (Table 1).

## 2. Syndromes

### 2.1. Circumferential Skin Creases Kunze Type

Circumferential skin creases Kunze type is a newly defined syndrome that is characterized by circumferential creases, cleft palate, typical face, intellectual disability, and growth retardation.

Congenital circumferential skin creases (or folds) are generalized folding of redundant skin that leads to multiple, symmetrical, ring-shaped creases of the limbs, trunk, and neck. Also, the term “Michelin tire baby syndrome” was used to describe these patients because of a resemblance of these patients to the logo of the French tire manufacturer [1]. The pathophysiology of these skin creases is not clear. It may result from a variety of underlying abnormalities including nevus lipomatosus, smooth muscle hamartoma, fragmented elastic fibers with smooth muscle hamartoma or the skin may be normal histopathologically [2–5].

Congenital circumferential skin creases may be an isolated anomaly or may be associated with multiple congenital anomalies and developmental abnormalities [3]. The HITCH syndrome (hearing impairment, undescended testis, circumferential skin creases, and mental handicap) is one of the known associations of congenital circumferential skin folds [6]. When they occur as an isolated anomaly, an autosomal dominant inheritance is often seen and patients show a tendency of gradual resolution with time.

In 2011, Wouters et al. reported two patients with congenital circumferential skin folds with multiple congenital anomalies and when they reviewed the literature, they noticed six patients with an almost identical clinical phenotype [7]. The common characteristics of these eight patients (6 male, 2 female) included circumferential skin folds affecting predominantly the upper and lower limbs, cleft palate, similar facial appearance, and delay of growth and development. The common facial features included short and upslanting palpebral fissures, epicanthal folds, microphthalmia, broad nasal bridge, microstomia, micrognathia, low-set posteriorly rotated ears with upturned ear lobes, and a flat midface. Except for two patients with a small head circumference, growth parameters were normal in all of the patients at birth, but during infancy growth retardation was detected. Initial hypotonia and delayed cognitive functions were reported for most of the patients. The hypoplastic corpus callosum and dilated lateral ventricles were the most frequent findings on brain imaging. Genital anomalies such as cryptorchidism, hypospadias, and hypoplastic scrotum were detected in four of six male patients. The fathers of two patients were affected by circumferential skin creases during the first years of life without any other malformation; gradually their skin creases disappeared.

Wouters et al. propose the term “circumferential skin creases Kunze type” to describe these patients who are different from patients with “Michelin tire baby syndrome” in terms of accompanying features to the circumferential skin creases, including cleft palate, typical face, intellectual disability, and growth retardation [7].

### 2.2. Unusual Type of Pachyonychia Congenita or a New Syndrome

Pachyonychia congenita (PC) is a rare keratin disorder usually inherited in an autosomal dominant manner [21]. It is characterized by hypertrophic nail dystrophy and palmoplantar keratoderma and may also affect the oral mucosa, tongue, larynx, teeth, and hair [21, 22].

Two main subtypes of PC have been distinguished, the Jadassohn-Lewandowsky (PC type 1, PC-1) and Jackson-Lawler syndromes (PC type 2, PC-2). Mutations affecting one of the paired keratins of specialized epidermis have been found to be the molecular basis for both of these disorders. While the PC-1 is associated with mutations in K6a and K16, the PC-2 is associated with mutations in K6b and K17 [21]. PC-1 pachyonychia is associated with focal palmoplantar hyperkeratosis, follicular keratosis, and oral leukokeratosis. PC-2 pachyonychia is associated with natal teeth and multiple pilosebaceous cysts and also focal palmoplantar keratoderma, follicular keratosis, and hair abnormalities [23].

**Figure 1:**
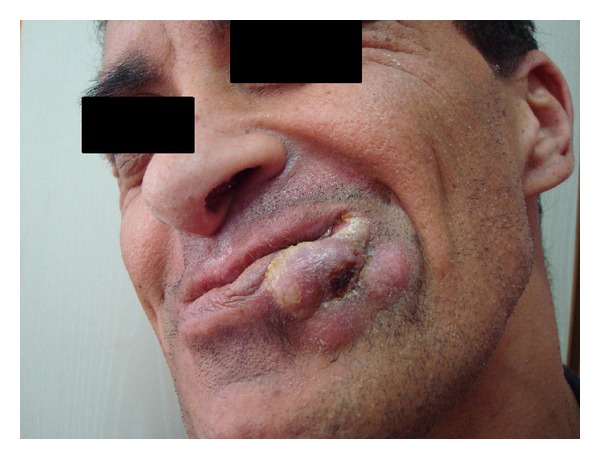
Squamous cell carcinoma of the lower lip mucosa.

In 2013, Gönül et al. reported a male patient with follicular hyperkeratosis, bilateral blepharitis, epidermal cysts localized to the axillae, painful focal palmoplantar keratoderma, oral leukokeratosis, complete loss of teeth, nails with proximal layering, and mild mental retardation. Laboratory investigations were within normal limits except for a deficiency of immunoglobulin A. Keratin gene (KRT6a, KRT6b, KRT16, and KRT17) mutations for PC were negative. After the patient received a removable dental prosthesis, squamous cell cancer which is unexpected in oral lesions of PC developed on the lower lip mucosa during follow-up (Figure 1) [8].

The characteristic nail changes of PC include prominent thickening of the nail bed and progressive distal elevation. In this patient, proximal layering of all the nails was unlike characteristic thickened dystrophic nails of PC. The authors suggested this case may be a new syndrome or an unusual new type of pachyonychia congenita because of this differing nail dystrophy, blepharitis, complete loss of teeth, and negative keratin gene mutations for pachyonychia congenita and developed squamous cell cancer on the oral leukokeratosis lesions [8].

## 3. Autoinflammatory Disease

### 3.1. Chronic Atypical Neutrophilic Dermatosis with Lipodystrophy and Elevated Temperature (CANDLE) Syndrome

**Figure 2:**
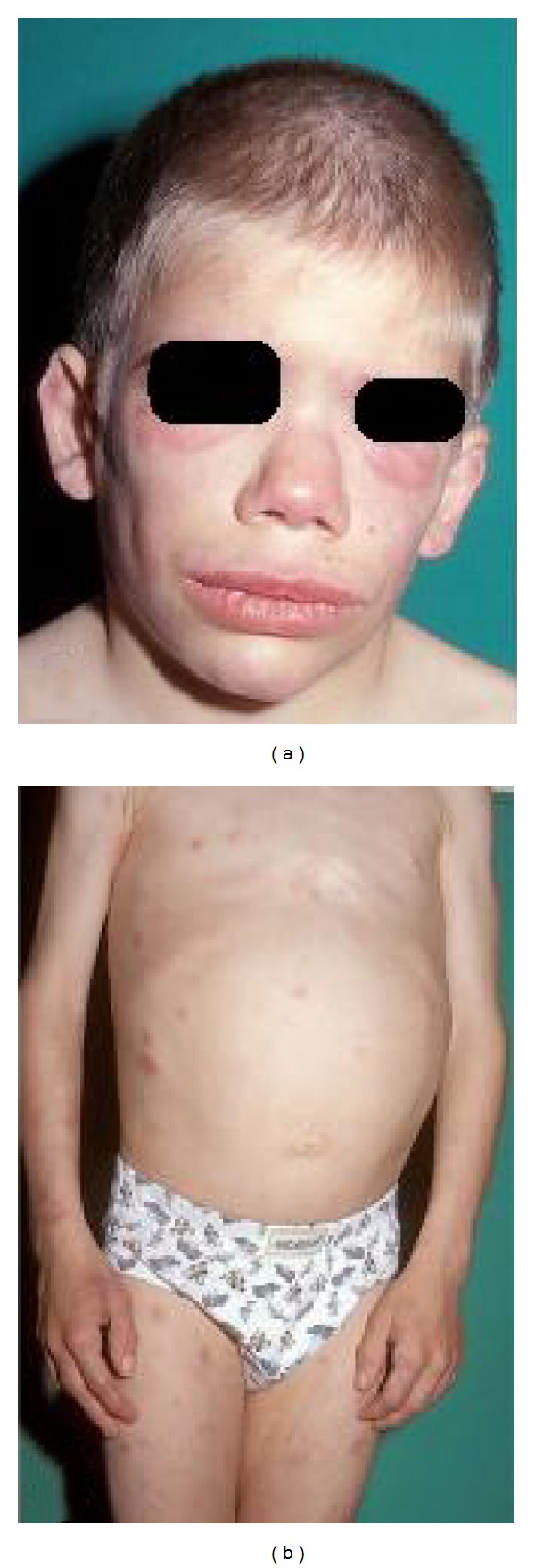
(a) Thick lips, persistent violaceous erythema of the eyelid, and decreased fatty tissue of the face. (b) Annular violaceous plaques on trunk and limbs, protuberant abdomen. Republished from [9] with permission from Elsevier.

Chronic atypical neutrophilic dermatosis with lipodystrophy and elevated temperature (CANDLE) syndrome refers to a newly described autoinflammatory disease which consists of early onset recurrent fever, characteristic skin lesions such as annular violaceous plaques, persistent violaceous eyelid and lip swelling, delayed physical development, progressive lipodystrophy, hepatomegaly, and a range of visceral inflammatory manifestations (Figure 2). The hallmark histological examination of skin lesions is atypical or immature myeloid infiltrates.

CANDLE syndrome was first described in four patients having unique clinical, histopathological, and laboratory features in 2010 by Torrelo et al. The patients were 8, 10, 14, and 2 years old. The fourteen- and two-year-old patients were sisters. Features common to all patients were early disease onset (in the first year of life), daily or recurrent fever with a poor response to NSAIDs, delayed physical development (low weight and height), recurrent annular violaceous skin plaques, typical facial appearance with violaceous swollen eyelids and thick lips, progressive lipodystrophy, hepatomegaly, and arthralgia without arthritis. Two patients had conjunctivitis, nodular episcleritis, prominent abdomen, aseptic meningitis, and splenomegaly. Ear and nose chondritis, epididymitis, parotitis, nephritis, otitis, interstitial lung disease, lymphadenopathy, acanthosis nigricans, and hirsutism were all seen in only one patient. Laboratory abnormalities included the evidence of systemic inflammation with increased erythrocyte sedimentation rate (ESR) and C-reactive protein (CRP) and mild elevation of hepatic transaminase levels and hypochromic anemia in all patients. Increased platelet counts, increased triglyceride levels, and basal ganglia calcifications were detected in two patients. There was no evidence of underlying malignancy, including blood or bone marrow dyscrasias. Histopathologically, skin biopsy specimens of all patients demonstrated an inflammatory reaction comprising both mature neutrophils and some mononuclear cells without vasculitis and perivascular and interstitial infiltrates of atypical mononuclear cells of myeloid lineage which was confirmed by immunohistochemical stains. Different treatments including NSAIDs, systemic steroids, dapsone, methotrexate, azathioprine, cyclosporine, and biological agents were administered with no or minimal benefit. The authors made a detailed evaluation of these four patients. Inherited autoinflammatory disorders such as chronic infantile neurologic cutaneous and articular syndrome (NOMID), familial Mediterranean fever, tumor necrosis factor receptor-associated periodic syndrome (TRAPS), and hyperimmunoglobulin D syndrome; well-known lipodystrophy syndromes such as acquired partial lipodystrophy of Barraquer-Simons, congenital generalized lipodystrophy of Berardinelli-Seip, familial partial lipodystrophy and leprechaunism; and Sweet syndrome, leukemia cutis, annular erythema of infancy, erythema annulare centrifugum, familial annular erythema, erythema chronicum migrans of Lyme disease, eosinophilic cellulitis, neonatal lupus erythematosus, annular urticaria, erythema multiforme, and erythema gyratum repens were considered in the differential diagnosis and all were excluded based on clinical and histological findings, in addition to laboratory and genetic testing. The authors suggested that these four patients seem to have a syndrome which has not been recognized yet and proposed the acronym CANDLE syndrome for this new and likely genetic disorder [9]. In 2011, one more case of CANDLE syndrome was reported by Ramot et al. [24]. A recent genomic analysis of nine patients showed mutations in the proteasome subunit *β* type 8 (PSMB8), with five patients having been previously reported and four new patients [25]. Newly described patients with CANDLE syndrome in the future will help us to understand the pathogenesis of this disorder and to find a more effective therapy.

### 3.2. Pyoderma Gangrenosum, Acne, and Hidradenitis Suppurativa (PASH) Syndrome

**Figure 3:**
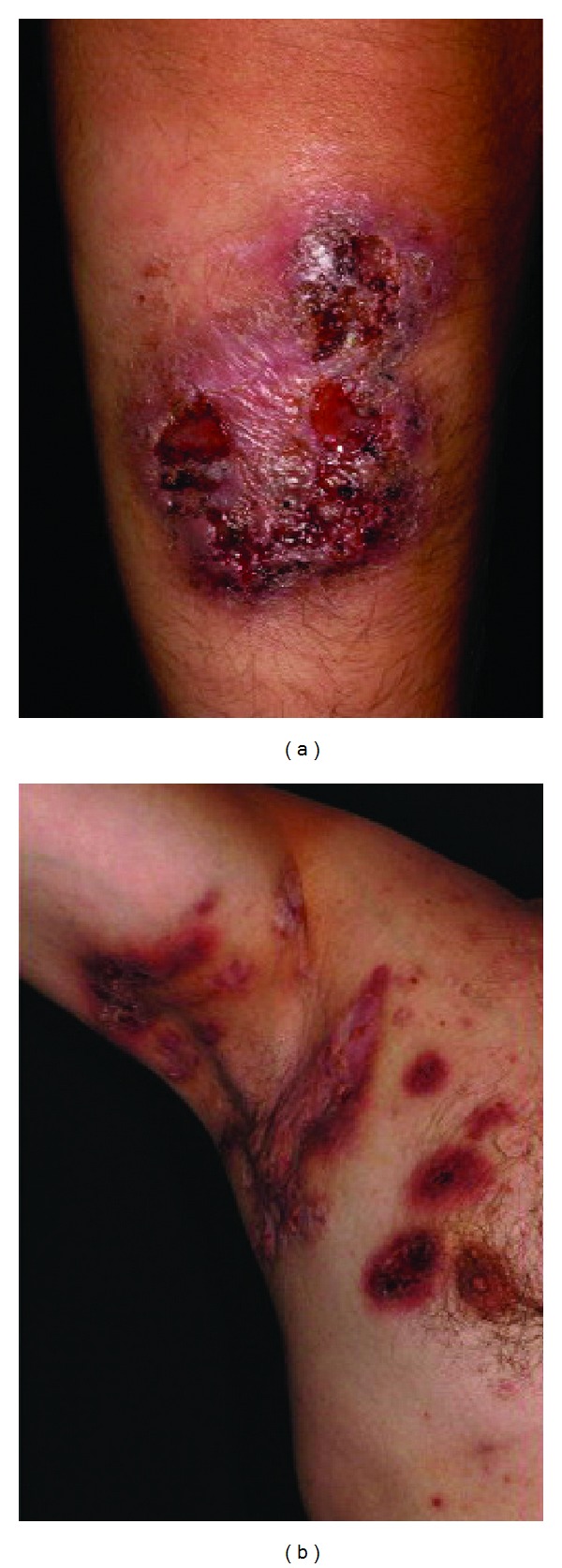
(a) Multilocular pyoderma gangrenosum on lower leg. (b) Suppurative hidradenitis in axilla. Republished from [10] with permission from Elsevier.

PASH syndrome is a recently described autoinflammatory syndrome, which is characterized by pyoderma gangrenosum (PG), acne, and hidradenitis suppurativa (HS). Braun-Falco et al. [10] named PASH syndrome first in the literature in 2012. They described two male patients, who had ulcerations, cystic acne lesions (one patient had active and the other had medical history), and draining sinus and abscesses. First patient was a 34-year-old man who had severe cystic acnes on his face and back and had a draining sinus and abscesses in both axillae for 7 years. He also had painful ulcerations on his arms and lower legs, which expanded slowly over a six-month period (Figure 3). The patient was otherwise healthy. Dermatological examination revealed large inflammatory, suppurative, and scarring plaques with draining sinuses and abscesses in both axillae. On his chest, back, and lower extremities, several crusted ulcers and hemorrhagic scarring plaques up to a size of approximately 10 × 15 cm were seen. He had multiple depressed scars resulting from conglobate acne on his face and back. The second patient was a 44-year-old man who had 22 × 12 cm ulcer on the front, lower aspect of his left leg that was surrounded by a dusky red, inflammatory, undermined edge. The ulceration appeared after a minor trauma, which healed almost completely after an autologous split-skin grafting, but later recurred. Physical examination showed multiple inflammatory and scarring plaques with abscesses, fistulae, and keloidal scars in both the axillae and groin and along the large fold under his overhanging abdomen. His medical history was remarkable only for severe cystic acne that occurred between the ages of 17 and 18, which left behind multiple depressed scars on both cheeks. Histopathological examinations of ulcers were similar on both patients and consistent with PG [10].

The dermatologic signs of PG and acne with pyogenic sterile arthritis, PG, and acne (PAPA) syndrome have been defined as an autosomal dominant autoinflammatory syndrome caused by mutations in the proline-serine-threonine-phosphatase interactive protein 1 (PSTPIP1) gene [10, 26]. Severe HS occurs at large skin folds and lacks the intensive joint inflammation observed in these patients. These features are not seen in PAPA syndrome. Mutations in the PSTPIP1 gene, MEFV, NLRP3, and TNFRSF1A genes were investigated. No mutations could be detected, but an increased number of CCTG microsatellite repeats in the PSTPIP1 promoter region were found by Braun-Falco et al. These authors also reported that CCTG replications in the PSTPIP1 promoter region may have only a mild effect on the development of this rather distinct cutaneous inflammation [10]. Previously in the literature, such an increase has been observed in aseptic abscess syndrome reported in French patients [27]. Its characteristic presentations included deep splenic abscesses, fever, abdominal pain, and leukocytosis. Also, this disorder showed an association with inflammatory bowel disease in 66% of these patients. Skin manifestations include cutaneous abscesses and neutrophilic dermatoses, primarily PG and Sweet's syndrome, seen in 20% of the patients. Consequently, this increase in number of the CCTG motif in the PSTPIP1 promoter, which likely deregulates PSTPIP1 expression, may predispose to other forms of neutrophilic inflammation, and perhaps the location of the defect within the PSTPIP1 gene may influence the organ predilection. Namely, substitutions in the amino acids encoded by exons 10 and 11 would bring about pyogenic arthritis, whereas CCTG replications/duplications in the promoter region would lead to splenic abscesses and/or skin manifestations. There is no standard treatment of PASH syndrome. Partial response was achieved with IL-1*β* medication. This implies that IL-1*β* may have a role in PASH syndrome pathogenesis [10].

The coexistence of PG, acne, and HS may represent a new disease entity within the spectrum of autoinflammatory syndromes such as PAPA and aseptic abscesses syndrome. In contrast to these two disorders, PASH syndrome has a clear predilection for the skin and lacks arthritis and visceral involvement [10].

### 3.3. Pyogenic Arthritis, Pyoderma Gangrenosum, Acne, and Hidradenitis Suppurativa (PAPASH) Syndrome

PAPASH syndrome is an autoinflammatory syndrome clinically characterized by pyogenic arthritis, pyoderma gangrenosum (PG), acne, and hidradenitis suppurativa (HS) associated with mutations in the PSTPIP1 gene. Marzano et al. first described this syndrome in 2013 [11]. Authors reported a 16-year-old female patient who had large inflammatory ulcerated plaques with draining sinuses, abscesses, and keloidal scars in her axillae, the intermammary and inguinal folds, and the anogenital area. In her history, she had experienced chronic-relapsing abscesses in the major skin folds and mild acne for two years. After six months, multiple skin ulcers appeared on her back. Dermatological examination revealed 1.0 to 2.5 cm oval or roundish ulcers with vegetating features. Histological examination showed features consistent with PG. Oral methylprednisolone, 50 mg daily, and topical tacrolimus ointment, 0.1%, applied twice daily, were added to her dapsone regimen for the treatment of PG. Following the tapering of her methylprednisolone dose, the PG relapsed, and her arthralgia recurred. Most severely affected areas were nonaxial joints, particularly the knee, elbow, shoulder, and hands. Genetic studies evaluating exons 10 and 11 of the PSTPIP1 gene revealed a previously unreported c.831G → T nucleotide substitution, leading to the p.E277D missense mutation. Treatment with the IL-1 receptor antagonist anakinra (100 mg/d) was started and her clinical condition improved [11].

In 2012, Braun-Falco et al. [10] described the newly discovered PASH syndrome, which featured the triad of PG, acne, and SH. As distinct from PAPA (pyogenic sterile arthritis, PG, and acne) syndrome, no mutation was detected in the PSTPIP1 gene in PASH syndrome [10]. Authors described this new entity, which may be included in a spectrum of disorders that will be defined by their clinical presentations as well as their specific genetic mutations [11].

## 4. Tumors

### 4.1. Acquired Reactive Digital Fibroma

Acquired reactive digital fibroma is a rare, benign fibroblastic tumor of the digit. Plaza et al. defined this new entity in five patients in 2013 [12]. All of the patients were male and older than 54 years who had been of mixed race. Before the mass lesion in the digits developed, patients had a recent trauma history two to six weeks previously. The subungual or periungual regions of the thumb or the big toe were the most frequently observed areas for this fibroblastic tumor. The mass gradually increased in size and often had a further clinical progression of deficit of motion and function of the digits. A nontender, well-delineated, immobile nodular mass (2.5–3 cm) on the distal portion of the digits was observed on dermatological examination. A malignant appearing ulceration was found only on one patient. There were no signs of infection or constitutional symptoms such as fever, weight loss, or malaise. Radiologic studies demonstrated no bone involvement in any of the cases. Histologically, all lesions were composed of short fascicles of benign-appearing fibroblastic spindle cells with vascularized and somewhat myxoid stroma. There were no mitotic figures in the lesion. Most cases were located in the dermis; however, two cases focally involved the subcutaneous fat. The spindle cells expressed vimentin and in two cases CD34, supporting a component of dendritic fibroblastic cells as part of the lesion. All cases were negative for SMA (smooth muscle antibody), h-caldesmon, desmin, MSA (muscle specific antigen), Bcl-2, CD68, CD99, b-catenin, factor XIIIA, S100p, and EMA (epithelial membrane antigen). The morphologic appearance and the immunohistochemical profile of the cells are, thus, consistent with a fibroblastic proliferation. Fibro-osseous pseudotumor of the digit, parosteal nodular fasciitis (NF), fibroma of tendon sheath (FTS), superficial acral fibromyxoma, acquired digital fibrokeratoma, and cellular digital fibromas should be considered on the histologic differential diagnosis [12].

Fibro-osseous pseudotumor of the digit is a rare reactive fibroblastic/myofibroblastic proliferation with osseous differentiation [28]. The tumor most commonly affects women. The lesion develops in fingers and toes of young to middle aged adults [28, 29]. Usually, a well-delineated, predominantly dermal spindle cell lesion composed of mature and immature fibroblastic foci, which is associated with osteoid formation, were observed on histopathologic examinations. Nuclear atypia and mitotic figures can be seen in cellular areas [28]. Nodular fasciitis (NF), which should be considered in the differential diagnosis, is a rapidly growing lesion composed of benign fibroblastic tissue. NF is most commonly seen in young and middle-aged adults. The upper extremity and the hand are the typically involved areas and the feet are rare sites of involvement [30]. NF is differentiated from the acquired reactive digital fibroma by composing myofibroblastic cells, which exhibits coexpressed vimentin and smooth muscle-associated markers, such as muscle-specific actin, smooth muscle actin, h-caldesmon, and desmin. Also, FTS, superficial acral fibromyxoma, acquired digital fibrokeratoma, and cellular digital fibromas should be differentiated from an acquired reactive digital fibroma. Plaza et al. reported this unique disease to be a benign tumor with no risk of recurrence or malignant potential detected on a mean follow-up period of 8.6 years. Awareness of acquired reactive digital fibroma is important to prevent misdiagnosis and overtreatment of these small benign tumors [12].

### 4.2. Onychocytic Matricoma and Onychocytic Carcinoma

Onychocytic matricoma (OCM) and onychocytic carcinoma (OCC) are newly described onychogenic epithelial tumors [13, 14].

OCM refers to acanthoma of the nail matrix producing onychocytes. It was first described in five patients by Perrin et al. in 2012 [13]. It is a localized (monodactylous) longitudinal melanonychia which is slightly raised. It usually presents as pachymelanonychia which defines the two clinical features of the tumor. Pachyonychia indicates a localized thickening of the nail plate and melanonychia indicates its longitudinal pigmented band. OCM is composed of a basal compartment with a varying admixture of prekeratogenous cells and keratogenous cells. Endokeratinization originating in the deep portion of the tumor and nests of prekeratogenous and keratogenous cells in concentric arrangement are characteristic features of OCM. Three architectural patterns can be identified as acanthotic, acanthotic and papillomatous, and keratogenous. The main differential diagnosis of OCM is seborrheic keratosis. OCM clinically looks like irritated seborrheic keratosis but can be easily distinguished histologically [13, 31].

OCC was first described as malignant counterpart of OCM in one patient by Perrin et al. in 2013 [14]. Clinically it simulates onychomatricoma (OM) and verrucous Bowen disease but histologically OCC represents in situ malignant epithelial tumor exhibiting a distinct picture of onychocytic differentiation with signs of both nail matrical differentiation and nail plate differentiation.

Perrin et al. described OCC in a 52-year-old man with a 2-year history of a verrucous band of the lateral nail plate on the fourth finger of his left hand. Dermatological examination revealed a thickened lateral nail plate and marked curving associated with a yellowish discoloration without a black streak. A surgical excision was performed and histological examination showed a well-limited in situ epithelial tumor of the matrix and nail bed. Based on histomorphologic and immunohistochemical findings, OCC was distinguished from malignant OM and nail bed Bowen disease in the differential diagnosis.

The authors proposed a new type of nail band pattern concerning the peculiar thickening of the nail plate observed in OM, OCM, and OCC. This new type of nail band pattern is named as acquired localized (monodactylous) longitudinal pachyonychia (LLP). LLP melanonychia (pachymelanonychia) shows brown/black pigmentation and suggests OCM and rarely pigmented OM or OCC. LLP xantholeuconychia (xantholeucopachyonychia) shows a yellow to whitish appearance and suggests OM and rarely OCC [14, 31].

### 4.3. Infundibulocystic Nail Bed Squamous Cell Carcinoma

Infundibulocystic squamous cell carcinoma (ISCC) is a type of squamous cell carcinoma (SCC), which is considered to show infundibular differentiation. ISCC was first introduced by Kossard et al. in 2008 [32]. It has both well-differentiated and less differentiated forms. In recent studies, some cases showed benign cytological features with minimal basal atypia but were architecturally atypical so they were regarded as a low-grade carcinoma of a new dyskeratotic type of verrucous carcinoma (VC) or as a well-differentiated ISCC. Then the term infundibulocystic nail bed SCC (IBSCC) was proposed [31, 32]. IBSCC is clinically similar to classical subungual keratoacanthoma (SKA) but IBSCC is a larger tumor. The importance of its detection is that, unlike the deep classical SCC of this site, this particular lesion responds well to conservative excision to achieve a tumor-free margin, rather than amputation. The differential diagnosis of IBSCC includes proliferating tricholemmal cyst, proliferating epidermoid cyst, VC, and SKA. They are distinguished from IBSCC histopathologically [31].

### 4.4. Acral Histiocytic Nodules

Benign histiocytic proliferations are identified by their component cells and classified as either Langerhans cell histiocytosis or non-Langerhans cell (non-LC) histiocytosis. Non-LC histiocytoses, also known as non-X histiocytoses are a diverse group of disorders characterized by the accumulation of non-LC histiocytes. Many variants of the disease have been described displaying different characteristic clinical and histopathological features.

A possible new variant of non-X histiocytosis named “acral histiocytic nodules” has been described in only one patient in the literature. Patel et al. described a 57-year-old woman with a 14-year history of pale-yellow, firm, well demarcated multiple nodules which were 5–10 mm in size and located around the interphalangeal joints on both the extensor and flexural surfaces of hands. The number of lesions had gradually increased; some had enlarged and none resolved spontaneously. Some of the lesions had begun to be tender in time and lead to functional difficulties. The patient was otherwise well. Laboratory investigations showed no abnormalities. There was no bony involvement according to the plain radiographs and magnetic resonance imaging scans. On histological examination of an excisional biopsy of one lesion, well-circumscribed, hypercellular nodules extending into the dermis and subcutis with small amounts of fibrosis were observed. There was an abundance of lymphocytes and histiocyte-like cells with occasional multinucleated cells. There was no lipid or haemosiderin pigment, no cholesterol clefts and no ground-glass or foamy appearance of cells, mitoses were rare and no xanthoma cells were seen. Immunohistochemistry was positive for CD68 and focally for smooth-muscle actin, but negative for S100, CD34, desmin, epithelial membrane antigen and cytokeratin AE1/AE3. Based on clinical and histological findings the patient was considered to have non-X histiocytosis but was consistent with none of the recognized variants.

Benign cephalic histiocytosis, juvenile xanthogranuloma, generalized eruptive histiocytosis, reticulohistiocytomas, xanthoma disseminatum, Rosai-Dorfman disease, progressive nodular histiocytosis, xanthogranulomatous disease, and multicentric reticulohistiocytosis were excluded based upon the distinctive clinical, histological findings and extracutaneous features [15]. Benign cephalic histiocytosis, juvenile xanthogranuloma, and generalized eruptive histiocytosis are seen in the paediatric population [33, 34]. Reticulohistiocytomas are usually seen in young men as solitary lesions on the head and neck [15]. Xanthoma disseminatum has widespread involvement, sometimes affecting mucosal surfaces, and has an association with diabetes insipidus [35]. Rosai-Dorfman disease is seen in young people and large, painless bilateral cervical lymphadenopathy is a characteristic feature. Progressive nodular histiocytosis is a more severe and disfiguring disease with xanthomatous papules, deep nodules, and tumors. Xanthogranulomatous disease may have extracutaneous involvement [15]. Multicentric reticulohistiocytosis usually affects older women, cutaneous nodules on face and hands are found, and it is often associated with malignancy [36]. Finally, the patient was proposed to have a possible new variant of non-X histiocytosis and named “acral histiocytic nodules.” This new variant seems to have a benign course with no evidence of malignancy or extracutaneous involvement. Lesions leading to functional difficulties were excised in this patient as treatment.

## 5. Unclassified Disorders

### 5.1. Saurian Papulosis

Saurian papulosis is characterized by widespread, well-circumscribed, flat-topped polygonal papules covering most of the skin surface, but sparing the face, palms, and soles. The name of “saurian papulosis” is derived from the appearance resembling the skin of lizards, crocodiles, and dinosaurs [16].

**Figure 4:**
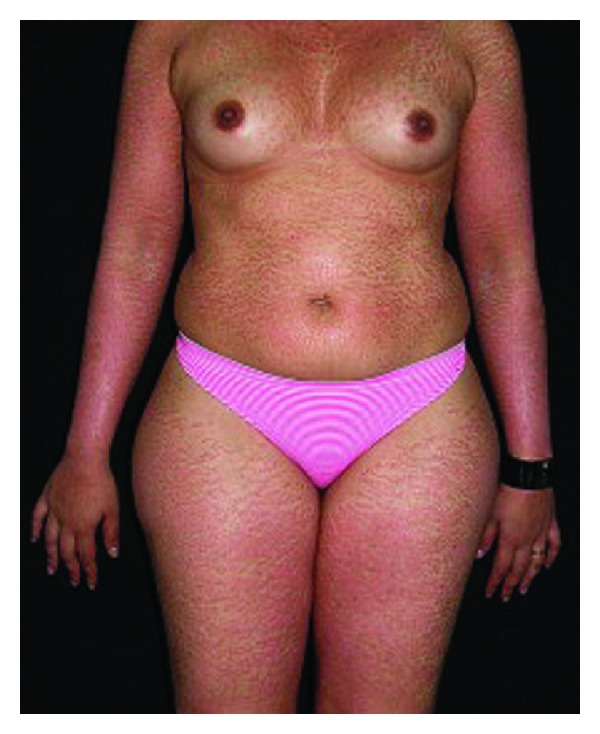
Generalized, flesh-colored hyperkeratotic papules involving trunk, upper and lower extremities. Republished from [16] with permission from Elsevier.

The disease was first described in 2013 by Molina-Ruiz et al. on two patients and one sibling with an asymptomatic skin eruption on the trunk and extremities [16]. One of these patients whose parents were consanguineous had skin eruption on flexural areas shortly after birth, resolving spontaneously over several months. After 14 years old, she developed a slowly progressive papular eruption covering most of the skin surface. The other patient whose parents were not consanguineous had lesions that appeared at the age of 62. Dermatological examinations revealed numerous, flesh colored or reddish, flat-topped, discrete polygonal papules with a fine scale, varying in size from 2 mm to 1 cm. The papules covered most of the skin surface and, in some areas of the trunk, they were arranged along the lines of cleavage, parallel to the ribs (Figure 4). There was no facial, mucosal, nail, or palmoplantar involvement; the teeth and hair were normal. The histopathologic findings demonstrated compact eosinophilic orthokeratotic hyperkeratosis overlying a slightly acanthotic epidermis. No parakeratosis was observed. The granular layer was spared and of normal thickness. A slight superficial, perivascular, lymphohistiocytic infiltrate was present in the underlying dermis. Immunohistochemical studies of these two patients showed diminished expression of connexin 43 in lesional skin compared with adjacent normal skin and controls [16].

The connexins are a family of proteins, which function as part of gap junctions in serving as cell-to-cell channels. They are expressed in several tissues such as brain, skin, and cochlea. Mutations in connexin genes may cause nonsyndromic sensorineural deafness. In addition, these mutations may result in variable dermatological features. Connexin gene mutation induced hereditary skin disorders include erythrokeratoderma variabilis, palmoplantar keratoderma associated with hearing loss, hydrotic ectodermal dysplasia (Clouston syndrome), and keratitis-ichthyosis-deafness [37, 38]. Oculodentodigital dysplasia syndrome is the only known dermatologic disorder related to connexin 43 dysfunction [39].

Saurian papulosis is thought to be another connexin-related disorder of epidermal keratinization and to have an autosomal recessive trait. Acquired ichthyosis, acrokeratosis verruciformis, epidermodysplasia verruciformis, flat warts, hyperkeratosis lenticularis perstans, ichthyosis vulgaris, keratosis lichenoides chronica, porokeratosis, pityriasis rubra pilaris, stucco keratoses, and waxy keratosis should be considered in the differential diagnosis [16]. Acrokeratosis verruciformis is an autosomal dominant genodermatosis usually presenting with skin color, multiple planar wart-like lesions, typically observed on the dorsum of the hands and feet. The forehead, scalp, flexures, and the oral mucosa are never affected. The papules show considerable hyperkeratosis, an increase in thickness of the granular layer, and acanthosis. In addition, there is frequent slight papillomatosis. There is elevation of the epidermis with a church spire appearance. The rete ridges are slightly elongated [16, 40]. Epidermodysplasia verruciformis (EV) is a rare genodermatosis with an autosomal recessive inheritance. Clinically, EV is characterized by the early onset of multiple flat warts, pityriasis versicolor-like lesions, and lesions resembling seborrheic keratosis. Histologically, EV is characterized by hyperkeratosis, hypergranulosis, and acanthosis. The most striking and characteristic feature is the presence of enlarged keratinocytes in the upper epidermis showing perinuclear halo and bluish cytoplasm [16, 41]. Hyperkeratosis lenticularis perstans is characterized by small erythematous or brownish hyperkeratotic papules distributed symmetrically on the limbs, particularly the dorsal feet and lower third of the legs. The etiology of hyperkeratosis lenticularis perstans is unknown. It may also be hereditary, with an autosomal dominant transmission. Histologically, the condition is characterized by hyperkeratosis, a thinning or absence of the granular layer, epidermal atrophy, and a band-like infiltrate in the upper dermis [42]. Waxy keratosis of childhood presents as well-demarcated keratotic papules secondary to abnormal keratinization. The lesions are usually localized on the trunk and proximal limbs. Histopathological examination revealed marked basophilic laminated hyperkeratosis, mild acanthosis, and papillomatosis [43].

Saurian papulosis has different clinical and histopathologic features from the other epidermal keratinization disorders [16].

### 5.2. Symmetrical Acrokeratoderma

Symmetrical acrokeratoderma is described as brown to black hyperkeratotic patches symmetrically distributed on the acral regions, especially on the wrists, ankles, and dorsum of the hands, fingers, and feet, but sparing palms and soles. In the English literature, it was first reported by Fan et al. in 2010, but actually it was first described in Chinese literature as “symmetric acral keratoderma” in 2008 [17]. To date, 243 cases have been reported in the Chinese and English literature [17, 44, 45].

**Figure 5:**
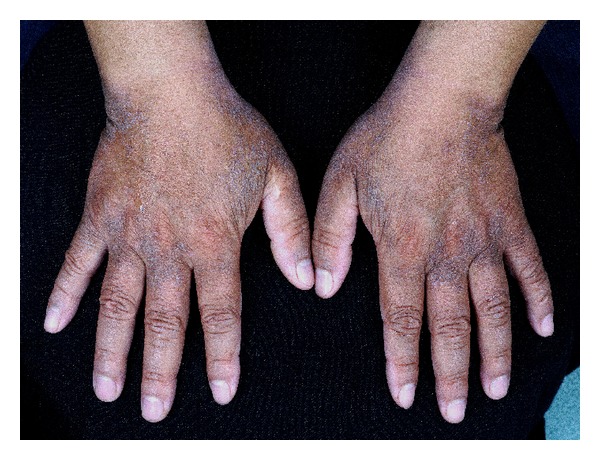
Brown to black hyperkeratotic patches on the back of both hands.

Symmetrical acrokeratoderma typically affects the young and middle aged men in China and characterized by brown to black hyperkeratotic patches symmetrically distributed on the acral regions, especially on the wrists, ankles, and dorsa of hands, fingers, and feet, but sparing the palms and soles (Figure 5). The lesions become whitish with mild swelling after 3–6 minutes with water immersion or sweating but recover gradually after drying. Typically, the condition worsens in summer and improves spontaneously in winter. Generally, no subjective symptoms were accompanied except mild itching [17, 44, 45].

Liu et al. reported one child whose skin lesions developed at two years of age and one patient with a family history, but most of the patients have no hereditary tendency [45]. Although some patients were reported to have a personal and/or family history of ichthyosis vulgaris, the relationship between ichthyosis vulgaris and symmetrical acrokeratoderma is poorly understood [45].

In some cases, direct examinations with potassium hydroxide were found to be positive and Malassezia was isolated from the lesions but it was thought to reflect normal skin colonization with no relationship to disease pathogenesis [17, 45].

Typical histopathologic features are hyperkeratosis, acanthosis, and superficial perivascular lymphohistiocytic infiltration. Electron microscopy examination of the immersed lesion demonstrates epidermal hyperkeratosis, spongiotic changes, and partial split of the desmosomes [17].

Symmetrical acrokeratoderma ought to be distinguished from acral acanthosis nigricans (acral acanthotic anomaly), palmoplantar keratodermas, aquagenic acrokeratoderma, focal acral hyperkeratosis, progressive symmetrical erythrokeratoderma, terra firma-forme dermatosis, and friction melanosis. Although acral distributions of hyperkeratotic lesions with normal palms and soles are common characteristics for both symmetrical acrokeratoderma and acral acanthosis nigricans, no seasonal and immediate postwater changes are seen in the latter [17]. Palmoplantar keratodermas are characterized by palmoplantar involvement with abnormal thickening of skin [17]. In aquagenic acrokeratoderma, translucent yellow or white papules appear on the palms and soles after brief immersion in water. Different from terra firma-forme dermatosis, the dirt-like lesions cannot be removed by alcohol swabbing in symmetrical acrokeratoderma [45].

No effective therapy has been reported to date. Lesions can be controlled with application of topical tretinoin and glucocorticoid ointment but recurrences are seen frequently. Limited or temporary improvements were reported with oral isotretinoin, ketoconazole, nicotinamide, vitamin A, vitamin B6, vitamin D, and vitamin E [17]. Avoidance of exacerbating factors, reduction of sweating and water immersion may be beneficial. Daily use of emollients should be recommended [45].

### 5.3. Confetti-Like Macular Atrophy

**Figure 6:**
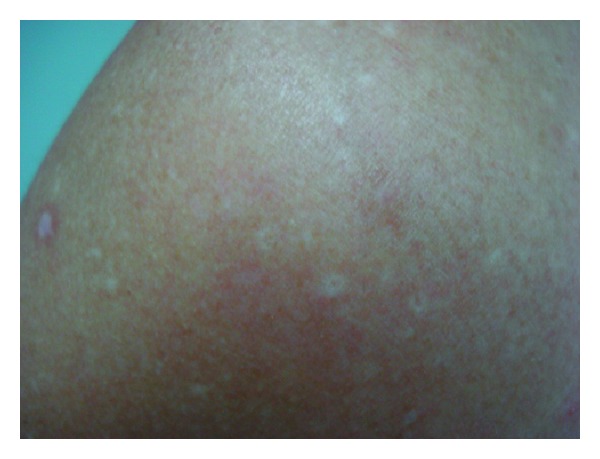
Multiple, confetti-like, atrophic, hypopigmented, flat macules on the upper trunk.

Confetti-like macular atrophy (CMA) is characterized by hypopigmented, atrophic macules at the same level with surrounding healthy skin with histopathological features that resemble both atrophoderma and anetoderma. It was first described by Aksoy et al. in 2009 in two patients with similar clinical and histopathological features [18]. Both of the patients were women, 34 and 42 years old, and presented with the complaint of white spots present for 13 and five years, respectively. The lesions appeared spontaneously without preceding inflammatory lesions and become more apparent after sun exposure. In physical examination there were multiple, 2–5 mm, discrete, atrophic, shiny, hypopigmented, perifollicular and nonfollicular, flat macules on the upper trunk and the extensor surface of the upper limbs (Figure 6). Lesions were at the same level with the surrounding healthy skin. Histopathological examination revealed an atrophic epidermis, a decrease of melanocytes and pigment content in the basal layer, perivascular mononuclear infiltration in the upper dermis, variable elastic tissue loss and fragmentation in the upper dermis, and disorganized, hyalinized, and coarse collagen bundles in the middle and lower dermis. No hyphae or spores were detected in periodic acid schiff (PAS) stain examination.

These lesions were somewhat different from all known causes of hypopigmented, atrophic macules clinically and histopathologically. Anetoderma, also known as macular atrophy, is characterized by small, atrophic, herniated papules. Anetoderma has similar histopathological features with the loss and fragmentation of elastic fibers in the dermis but it has a different clinical appearance with multiple, round to oval, well defined, finely wrinkled, herniated, skin colored papules [46]. Atrophoderma of Pasini and Pierini is characterized by single or multiple, sharply demarcated, gray or brown, slightly depressed, nonindurated patches of varying sizes. It may have similar histopathological features of epidermal atrophy, perivascular and interstitial lymphohistiocytic infiltration, homogenization and clumping of collagen bundles but unlike confetti-like macular atrophy, elastic tissue abnormalities are not present [47]. Although atrophoderma elastolyticum discretum has similar histopathological features of thickening of collagen fibers in the reticular dermis and loss of elastic tissue fibers in papillary and reticular dermis, the clinical appearance is quite different with 3–6 cm, depressed, pink-brown colored, atrophic plaques [48]. In atrophying tinea versicolor, epidermal colonization with hyphae or spores of Pityrosporum is seen histopathologically [49]. The small porcelain white, shiny, round macules of lichen sclerosus et atrophicus may resemble lesions of CMA clinically, but lichen sclerosus et atrophicus has different histopathological features [50].

### 5.4. Skin Spicules

Hyperkeratotic spicules is a rare cutaneous disorder of unknown origin defined by the presence of multiple foci of circumscribed hyperkeratosis with a column of keratotic material protruding from the stratum corneum [51]. Previously in the literature, different names were used for hyperkeratotic spicules such as filiform hyperkeratosis, parakeratotic horns, follicular hyperkeratosis, and multiple minute digitate hyperkeratosis [52]. Hyperkeratotic spicules can be associated with hypovitaminosis A, chronic renal failure, Crohn's disease, lymphoma, paraproteinemia, Sezary syndrome, multiple myeloma, dysglobulinemia, and other malignant diseases. Goldstein reported the first case of digitate hyperkeratotic lesions entitled with multiple minute digitate hyperkeratosis in 1967 [53]. These lesions are usually characterized by yellowish keratotic spicules predominantly localized on the face, nose, scalp, and, occasionally, the lumbar area [54].

Also, Hu et al. reported an unusual variant of the sign of Leser-Trélat in a patient with a cutaneous low-grade B-cell lymphoproliferative disorder and intertriginous skin spicules in 2009 [19]. Authors reported a middle aged Caucasian man who had an asymptomatic firm, well-demarcated plaque on the left side of his abdomen. The lesion was slowly enlarging for seven years. Dermatological examination showed 4.3 × 2.2 cm erythematous plaque on the left side of his abdomen and several hundred 1 to 2 mm white hyperkeratotic, perifollicular papules located primarily in the axillae and popliteal fossae that could not be removed manually. The results of histochemical and molecular genetic studies established the diagnosis of a cutaneous low-grade B-cell lymphoproliferative disorder specimen taken from abdominal plaque. The skin spicules were also biopsied, with the purpose of ruling out a cutaneous deposition process that has been reported in the literature in association with plasma cell dyscrasias. The histological examination revealed small seborrheic keratoses without a depositional process. After the abdominal plaque had been removed, the number of skin spicules was decreased. After a six-month period, the patient had an increasing number of skin spicules and a new asymptomatic smooth firm pink nodule was noted on the right upper aspect of his back [19].

The authors reported that the association between the skin spicules and the cutaneous lymphoma might be developed due to a paraneoplastic phenomenon. It was also emphasized that the skin spicules may represent a variant of the sign of Leser-Trelat [19]. The sign of Leser-Trelat was first described in 1890 as the sudden eruption of multiple seborrhoeic keratoses, or an increase in the number and size of existing seborrhoeic keratoses, associated with an underlying malignancy. The Leser-Trelat sign is most frequently associated with carcinomas of the stomach, lung and larynx, rectum, breast, and pancreas, mycosis fungoides, Sezary syndrome, and neurofibrosarcoma [55]. Although the histopathology of hyperkeratotic spicules usually showed focal columns of orthokeratotic or parakeratotic hyperkeratosis with homogenous compact eosinophilic inclusions, Hu et al. had found the histologic morphology of skin spicules resembled minute seborrheic keratosis. The authors explained the white church spire-like skin spicules as clinically distinct from the classic “stuck-on”-appearing waxy surfaces of tan- and brown-colored seborrheic keratoses, represents an unusual variant of the sign of Leser-Trelat [19].

### 5.5. Erythema Papulosa Semicircularis Recidivans

**Figure 7:**
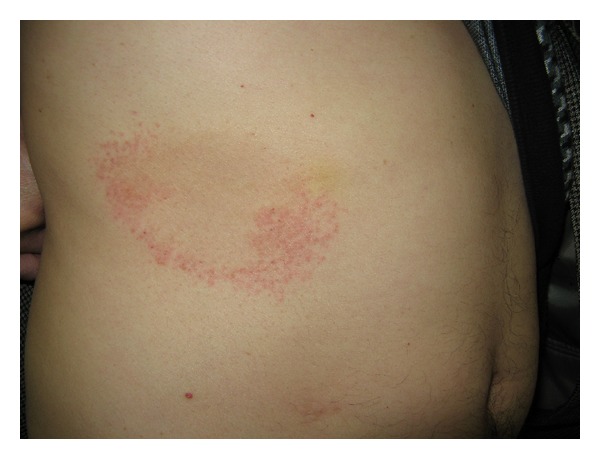
Large semicircular erythematous plaque with central clearing and multiple bright red papules studded on the border.

Erythema papulosa semicircularis recidivans is a new entity characterized by centrifugally expanding semicircular erythematous plaques that recur every year during the warm seasons. In 2012, Song et al. [20] reported nine patients with similar cutaneous findings of relapsing and centrifugally expanding papuloerythematous eruption. All of the patients were young and middle-aged men between 24 and 39 years of age living in Chongqing or in Sichuan province. They were presented with one to many large semicircular erythematous plaques with peripheral red elevated papular border (Figure 7). The lesions usually started at the beginning of summer, expanded centrifugally to involve large areas on the flank, anterior trunk, back, and thigh, gradually disappeared at the end of autumn, and recurred every year in warm seasons. Exacerbation of lesions after excessive sweating occurred frequently and mild pruritus was generally accompanied with the skin rash. Routine laboratory examinations of the patients were within normal limits. Skin-prick testing with a common airborne and food allergy series and patch testing with Chinese baseline series of contact allergens showed negative results. Histopathologic examination of tissue samples taken from the edges of skin lesions revealed superficial perivascular dermatitis with mild hyperkeratosis, slight edema of the papillary dermis, and superficial sparse perivascular lymphocytic infiltration.

The authors used the term “erythema papulosa semicircularis recidivans” to describe the suspected new dermatose which has distinct characteristics from other well-described figurate erythemas. Erythema annulare centrifugum is a different disease with erythematous edematous borders and has smaller eruptions with no sex or seasonal predilection [20, 56]. Erythema migrans is an initial cutaneous manifestation of Lyme disease caused by Borrelia burgdorferi spirochetes. It is characterized by slowly migrating erythema or annular plaques. Histopathologically a superficial and deep lymphohistiocytic infiltrate with eosinophils and plasma cells are seen and spirochetes are detected in the skin. Also, anti-Borrelia antibodies may be observed [56]. Other described figurate erythemas are also excluded on the basis of clinical history, clinical, laboratory, and histopathological findings.

Only symptomatic relief of pruritus was achieved by oral antihistamines, topical corticosteroid, and coal tar creams but progression of lesions could not be prevented. Lesions and symptoms gradually disappeared under treatment at the end of Autumn. Authors reported spontaneous resolution of disorder after 2 to 5 relapses that occurs every year in warm seasons [20].

## Figures and Tables

**Table 1:** Clinicopathological findings of new described dermatological disorders.

Disease	Author	Publication date	Number, sex, and age, y	Clinics	Pathology	Accompanying signs or disease
Circumferential skin creases Kunze type	Wouters et al. [7]	2011	8, 6 M/2 F, NR	Circumferential creases, cleft palate, typical face, intellectual disability, and growth retardation	Thickened, closely packed collagen bundles in deep dermis; normal distribution of elastic fibers	None

Unusual type of pachyonychia congenita or a new syndrome	Gönül et al. [8]	2013	1, M, 30	Follicular hyperkeratosis, bilateral blepharitis, epidermal cysts localized to the axillae, painful focal palmoplantar keratoderma, oral leukokeratosis, complete loss of teeth, nails with proximal layering, and mild mental retardation	—	Deficiency of immunoglobulin A and squamous cell cancer

CANDLE syndrome	Torrelo et al. [9]	2010	4, 1 M/3 F, 2–14	Early onset recurrent fever, annular violaceous plaques, persistent violaceous eyelid and lip swelling, delayed physical development, progressive lipodystrophy, hepatomegaly, and a range of visceral inflammatory manifestations	An inflammatory reaction comprising both mature neutrophils and some mononuclear cells without vasculitis and perivascular and interstitial infiltrates of atypical mononuclear cells of myeloid lineage	Arthralgia, conjunctivitis, nodular episcleritis, epididymitis, ear and nose chondritis, prominent abdomen, aseptic meningitis, parotitis, interstitial lung disease, nephritis, otitis, acanthosis nigricans, and hirsutism. Increased ESR and CRP, hypochromic anemia, increased platelet counts, elevated AST and ALT, increased triglyceride levels, and basal ganglia calcifications

PASH syndrome	Braun-Falco et al. [10]	2012	2, M, 34, 44	Pyoderma gangrenosum, acne, and suppurative hidradenitis, lacked any flare of arthritis. No mutation in PSTPIP1	Necrotizing suppurative and focally fibrosing dermatitis with severe neutrophilic infiltration, consistent with PG	None

PAPASH syndrome	Marzano et al. [11]	2013	1, F, 16	Pyogenic arthritis, pyoderma gangrenosum, acne, and hidradenitis suppurativa. Mutations in the PSTPIP1	Consistent with PG	None

Acquired reactive digital fibroma	Plaza et al. [12]	2013	5, M, 54–68	2–6 weeks after the injury a nontender, well-delineated, immobile nodular mass on the distal portion of the digits appeared	Composed of short fascicles of benign-appearing fibroblastic spindle cells with vascularized and somewhat myxoid stroma. There were no mitotic figures in the lesion	None

Onychocytic matricoma	Perrin et al. [13]	2012	5	Slightly raised localized longitudinal melanonychia (pachymelanonychia)	Endokeratinization originating in the deep portion of the tumor and nests of prekeratogenous and keratogenous cells in concentric arrangement	None

Onychocytic carcinoma	Perrin et al. [14]	2013	1, M, 52	2 years of history of a verrucous band of the lateral nail plate on the fourth finger of left hand and marked curving with a yellowish discoloration	Well-limited in situ epithelial tumor of the matrix and nail bed	None

Acral histiocytic nodules	Patel et al. [15]	2012	1, F, 57	Pale-yellow, firm, well demarcated multiple nodules which were 5–10 mm in size and located around the interphalangeal joints on both the extensor and flexural surfaces of hands. The number of lesions had gradually increased; some had enlarged and none had resolved spontaneously	Well-circumscribed, hypercellular nodules extending into the dermis and subcutis with small amounts of fibrosis were seen. Abundance of lymphocytes and histiocyte-like cells with occasional multinucleated cells. No lipid or haemosiderin pigment, no cholesterol clefts, and no ground-glass or foamy appearance of cells; mitoses were rare and no xanthoma cells were seen. CD68 positivity and S100 negativity	None

Saurian papulosis	Molina-Ruiz et al. [16]	2013	2, 1 F and 1 M, 27, 82	Widespread, symmetric erythematous, flat, discrete papules with a polygonal shape and fine scaling resembling the skin of lizards, crocodiles, and dinosaurs. No facial, mucosal, nail, or palmoplantar involvement. The teeth and hair were normal	Well-demarcated areas of compact eosinophilic orthokeratotic hyperkeratosis overlying a slightly acanthotic epidermis	None

Symmetrical acrokeratoderma	Fan et al. [17]	2010	29, 25 M and 4 F, 9–54	Symmetric, brown hyperkeratotic patches on the acral parts of the extremities. Worsened in summer and improved spontaneously in winter, becoming whitish with mild swelling after 3–6 minutes of water immersion or sweating	Hyperkeratosis, acanthosis, and superficial perivascular lymphohistiocytic infiltration	None

Confetti-like macular atrophy	Aksoy et al. [18]	2009	2, F, 34, 42	2–5 mm, hypopigmented, atrophic macules at the same level with surrounding on the upper trunk and the extensor surface of the upper limbs	Histopathological features that resemble both atrophoderma and anetoderma	None

Skin spicules	Hu et al. [19]	2009	1, M, 43	Several hundred 1 to 2 mm white hyperkeratotic, perifollicular papules located primarily in the axillae and popliteal fossae	Mild papillary epidermal acanthosis with hyperkeratosis, consistent with early seborrheic keratosis	Cutaneous marginal zone B-cell lymphoma

Erythema papulosa semicircularis recidivans	Song et al. [20]	2012	9, M, 24–39	Centrifugally expanding semicircular erythematous plaques recurring every year in warm seasons. Exacerbating after excessive sweating, mild pruritus was generally accompanied, resolution of disorder after 2–5 relaps	Superficial perivascular dermatitis with mild hyperkeratosis, slight edema of the papillary dermis, and superficial sparse perivascular lymphocytic infiltration	None
